# Seeding Stress Resilience through Inoculation

**DOI:** 10.1155/2016/4928081

**Published:** 2016-01-05

**Authors:** Archana Ashokan, Meenalochani Sivasubramanian, Rupshi Mitra

**Affiliations:** School of Biological Sciences, Nanyang Technological University, Singapore 637551

## Abstract

Stress is a generalized set of physiological and psychological responses observed when an organism is placed under challenging circumstances. The stress response allows organisms to reattain the equilibrium in face of perturbations. Unfortunately, chronic and/or traumatic exposure to stress frequently overwhelms coping ability of an individual. This is manifested as symptoms affecting emotions and cognition in stress-related mental disorders. Thus environmental interventions that promote resilience in face of stress have much clinical relevance. Focus of the bulk of relevant neurobiological research at present remains on negative aspects of health and psychological outcomes of stress exposure. Yet exposure to the stress itself can promote resilience to subsequent stressful episodes later in the life. This is especially true if the prior stress occurs early in life, is mild in its magnitude, and is controllable by the individual. This articulation has been referred to as “stress inoculation,” reminiscent of resilience to the pathology generated through vaccination by attenuated pathogen itself. Using experimental evidence from animal models, this review explores relationship between nature of the “inoculum” stress and subsequent psychological resilience.

## 1. Stress and Stress Inoculation

Stress is a nonspecific response of the body to any demand placed by external environment or internal metabolic milieu [1]. The stress response itself cannot be eradicated or evaded for very long because these challenges are inevitable aspects of life. The concept of stress was first introduced in the work of Selye [1], who observed that individual animals exhibited a “general adaptation syndrome” when confronted with a variety of perturbations. He argued for a common bodily mechanism that was invoked during these challenging episodes and termed it “stress,” further remarking that the stress “suffers from the mixed blessing of being too well known and too little understood” [1]. In due course of time psychological dimensions were included in the repertoire of stress syndrome, including emotional and cognitive facets. In recent decades, relationship between stress and predisposition to mental disorders has gained mounting importance [2]. This renewed focus is borne out by reclassification of stress and trauma related disorders as an identifiable mental disorder in recent diagnostic manual [3]. The clinical interest is paralleled by increasing knowledge about neural and endocrine underpinnings of this process. In this regard, feedback loops between adrenal hormones and discrete brain regions have been extensively described and analysed (succinctly reviewed in [4–7]).

Despite its negative connotations, stress has an adaptive value in that it promotes homeostasis. Exposure to stressful events that are not devastating, yet challenging enough to provoke emotional instigation and cognitive processing, might nurture successful coping with subsequent stressors. Thus exposure to prolonged unpredictable and uncontrollable stress induces long-term neurological impairment, but exposure to moderately stressful and controllable events seems to increase efficacy of regulating future stress response. This phenomenon has been referred to as “stress inoculation” [8]. Rhetorically, stress inoculation is reminiscent of protection from a pathology afforded by prior inoculation with attenuated pathological agent. Just as vaccination by a dead or weakened pathogen enables the body to mount a long-lasting immune response, exposure to moderate amount of stress enables organisms to effectively cope with future stressors. Although this hypothesis has great potential, it remains relatively understudied at present.

The effects of stress on health outcomes often vary in a nonlinear manner with severity of the stress, roughly corresponding to an inverted U shaped reaction norm [9–11]. When starting from a low baseline, moderate amount of stressful challenge enhances both short-term and long-term health outcomes. With successive increase in severity, stress leads to diminished health leading to an inverted U relationship between stress and health parameters. The idea of inverted U shaped reaction norm was first formalized to explain relationship between arousal levels and strength of discrimination based learning [12]. The concept has been subsequently used to empirically explain variation in behaviour with respect to different kinds and strengths of stressor. A few studies have also demonstrated this relationship within a single experimental design. For example, rats swimming in colder water experience greater secretion of stress hormones (16°C > 19°C > 25°C) when undergoing training for spatial learning in radial arm water maze. Animals trained at moderate stress levels of 19°C perform fewer errors compared to those trained at either 16°C or 25°C [13]. Similarly direction of the corticosterone influence on hippocampal primed burst potentiation is dependent upon the concentration of this stress hormone [14]. Briefly, primed burst potentiation utilizes electrical stimulation that mimics pattern of endogenous activity of hippocampal neurons, leading to long-lasting increase in synaptic strength [15]. Low to medium corticosterone levels are positively correlated with primed burst potentiation, representing ascending part of the inverse U. At higher concentration, corticosterone levels become negatively correlated with electrophysiological potentiation, reflecting descending part of the curve.

Various factors influence the shape and inflexion point of such inverted U curve. These include age, sex, and predictability of the stress [16–19]. Very importantly, these factors also include degree, nature, and developmental timing of the historical stress exposure [20]. These factors are capable of shifting the curve from its general course towards either stress pathologies or stress resilience [9]. The essence of stress inoculation thus lies in the idea that prior stress exposure can induce resilience to later stress, if the prior stress is of optimal degree and provided at crucial stage of life.

## 2. State of Current Animal Models

Animals models are primarily used for stress research due to the methodological and ethical limitations involved in human studies [21]. A good animal model of stress inoculation will ideally comprise face validity, construct validity, and predictive validity (e.g., [22, 23]). In other words, an animal model of stress inoculation must contain elements that are analogous to human stress inoculation. It must comprise measurable endpoint that accurately reflects the unmeasurable theoretical construct of stress inoculation. Additionally, animal model in question must be able to prospectively predict strength of the inoculation. Beyond these, the animal models should be consistent and reproducible and have internal controls to measure influence of confound like locomotion or nutrition. Unfortunately, a consensus about the animal model that meets these criteria for stress inoculation remains elusive at present. A variety of inoculum stressors and correspondingly varied subsequent stressors to test the inoculation have been used. Further work is required to refine animal models with respect to both external and internal validity.

Within the limits of current animal models, researches on rodents and primates support the stress inoculation hypothesis and provide insight into its neurobiological mechanisms. Broadly, early life stress inoculation triggers broad developmental cascades that increase adaptation. For example, studies on male and female squirrel monkeys show that a brief intermittent maternal separation during early childhood enhances long-lasting and trait-like transformation in the multiple domains of adaptive functioning [24]. Young male and female monkeys presented with a moderate stressor in the form of periodic short maternal separation from postnatal week 17 to postnatal week 27 experienced acute distress during the separation periods manifested by agitation and temporary elevation in the stress hormone levels [25]. However later in life, at nine months of age, the same set of monkeys demonstrated lower anxiety and decreased stress hormone levels when compared to the control animals. Further these inoculated monkeys showed higher cognitive control when accessed at 1.5 years of age, higher curiosity when accessed at 2.5 years, and larger prefrontal cortex volume at 3.3 years of age, compared to the age matched noninoculated controls [25, 26]. These results suggest that engagement in new situations that require challenging but not overwhelmingly stressful experiences results in enduring effects that stimulate adaptation in cognitive, motivational, and socioemotional aspect of behaviour in primates.

Early developmental stages of an individual asymmetrically contribute to the shaping of resilience in later life. Several studies have demonstrated entrainment of adult behaviour as a consequence of stress during early development. For example, squirrels change their growth trajectories based on in utero exposure to stress [27], and early childhood stress results in earlier menarche in human females [28] and aversive conditioning during infancy blunts the strength of further conditioning in adulthood [29]. Congruently, majority of studies pertaining to stress inoculation provide initial stress in early life [30–34]. For instance, maternal separation in infant rodents since birth (continuously for 2 weeks, 3 hours per day) gives rise to hyperactive stress responses in the form of heightened stress hormone release [34]. However, intermittent brief separation of these pups from the mother results in adaptive endocrine responses characterizing resilient features [32]. Similarly in primates, when four-month-old squirrel monkeys are exposed to intermittent levels of the same form of maternal separation (ten sessions per week), it leads to emotionally stable responses under stressful situations and lowered release of stress hormones accompanied by more exploration of novel settings [24]. This presents an exemplary case of stress inoculation which is dependent on the developmental stage of an individual.

In should be noted that resilience in models involving maternal separation could result from behavioural change in either mother or the offspring. In other words, it is possible that early life stress promotes resilience because the separation changes maternal behaviour towards pups rather than stress inoculation of the pups themselves. For example, brief intermittent exposure to foot shock during infancy augments resilience. This is due to the increased maternal stimulation received after the rat pups are returned to their nest. This increased maternal stimulation has been shown to enhance stress regulation in pups that endure into their adulthood [30]. In contrast to rats, similar effects have not been observed in primates. For example, differences in the maternal behaviour did not correspond with differences in the development of arousal regulation in young monkeys [33]. Thus both the locus of initial behavioural change and the outcome can be idiosyncratically specific to the species being studied. This creates further challenge to create an animal model for studying stress inoculation in humans.

While early life has a long-lasting influence on the stress inoculation, several papers have also reported protective effects of preceding stress in adulthood. Thus stress inoculation in male mice by exposure to mild stress (noncontact interaction via resident intrusion) in adulthood leads to more emotionally stable response, lowered depressive like symptoms, and enhanced exploratory behaviour [35]. The same study also showed reduced secretion of stress hormones in response to repeated restraint. Similarly, exposure to three or more mild restraints before inescapable shock or three sessions of inescapable tail-shocks with intervening rest days attenuates development of learned helplessness in male rats [36]. In adult female squirrel monkeys, intermittent separation from group and introduction of novel group partners create a stress inoculation against future stressor [37]. This manifests as reduction in anhedonia and reduced activation of stress hormone axis when inoculated animals are exposed to a subsequent social separation. This generality of inoculation models across developmental stages, if reinforced by further studies, creates greater opportunity for use of this paradigm in the adulthood.

Gender presents an important consideration when interpreting effects of stress inoculation. Sexually dimorphic gonadal hormones robustly interact with brain and behaviour, including the stress response (reviewed in [38–41]). For example, major depression and anxiety disorder are more prevalent in women of reproductive age than corresponding male population [42]. In rats, chronic stress causes lesser anxiogenesis in females compared to males [43–46]. Biological substrates of gender dimorphism pertaining to stress remain understudied. Similarly, reasons for discordant direction of stress effects on humans and rodents are unclear at present.

## 3. Environmental Manipulation: A Potential Regulator for Hypothalamus-Pituitary-Adrenal Axis Tone

The degree of control that an animal has on a specific stressor plays a key role in defining whether the event will lead to ensuing vulnerability or resilience to the stress. Animals administered with unavoidable and unpredictable shock tend to develop exaggerated fear response, heightened anxiety, and deficits in active coping when faced with subsequent stressor [47], a phenomenon often referred to as learned helplessness [48]. However, animals that are given shock and are concomitantly given the ability to avoid them by modifying their behaviour do not develop learned helplessness [49, 50]. Similar effects have been observed in humans, whereby individuals previously inoculated by a controllable stress acquire resilience to a broader range of other subsequent stressors [51].

In terms of the endocrine activation, stress inoculation results in lower responsiveness and earlier termination of stress hormone secretion, while severe stress results in the opposite effect. For example, repeated maternal separation of rat pups results in lower expression of glucocorticoid receptors (GRs) in the hippocampus when these pups reach adulthood [52]. The hippocampal glucocorticoid receptors bind to circulating corticosterone (CORT), a stress hormone secreted by adrenal glands. The occupancy of these GRs then sends a negative feedback to the hypothalamus-pituitary-adrenal axis, thus terminating further stress hormone release. A reduction in glucocorticoid receptors leads to reduced efficacy of this negative feedback which then blunts the ability to terminate ongoing stress response. This example demonstrates that early environment can have long-lasting implications for future stress response.

CORT, the primary ligand for GR, is often measured to reflect ongoing stress response [53, 54]. Amount of circulating CORT in rodents (cortisol in primates) exhibits robust sensitivity to the environment [55]. For example, introduction of novelty in the environment causes increase in CORT, leading to an emotional arousal in the individual [56]. Modulations in the environment on an intermittent basis might lead to increase or decrease of CORT and when this is done in moderation, it can cause a shift in the threshold of the HPA activity for an individual [55, 57]. The resultant effect would reflect the shift in the inverted U shaped curve of stress response which corresponds to stress inoculation [9]. In brief, stress inoculation can shift dose response curve between future stress and performance leftward (greater performance at lower levels of future stress) and/or rightward (greater tolerance to higher level of stress) [9].

Thus both stress response and CORT are exquisitely responsive to the degree and nature of environmental changes. Interestingly, CORT itself causes differential neural plasticity in different brain regions. For example, chronic stress which raises the circulating CORT leads to neuronal atrophy in the hippocampus but hypertrophy in the basolateral amygdala (BLA) [58–60]. These contrasting effects are suggested to cause reduced memory performance due to chronic stress mediated by its hippocampal effects and increased anxiety mediated by its amygdalar effects. The dorsal region of the hippocampus is necessary for spatial learning and is directly linked to stress-induced memory deficits [61, 62]. Likewise, intra-BLA experimental manipulation suggests necessity and sufficiency of BLA changes for stress-induced anxiogenesis. For example, decreasing excitability of BLA neurons by overexpression of SK2 K^+^ channels simultaneously reduces stress-induced stress hormone secretion, anxiety, and BLA hypertrophy [63]. Similarly, rerouting of stress hormone signalling away from glucocorticoid receptors within BLA also reduces anxiety [64–66].

Several studies described above have used CORT levels as a proxy for HPA tone and implicitly as a proxy for stress responsiveness. Yet the relationship between CORT levels and HPA responsiveness is not linear. For example, effects of CORT depend on expression of GR and relative expression of GR/MR, in addition to specific brain regions expressing these receptors. It is noteworthy that an experimental change in ratio between MR and GR expression in the hippocampus can drastically change memory [67, 68]. Similar manipulation in the amygdala reduces anxiety and future endogenous CORT release [64]. This suggests that effects of CORT on brain and behaviour are dependent on expression level of receptors and type of the central receptors (GR and MR) available. The importance of the central receptors is further supported by the observations that stress in rodents and monkeys can downregulate hippocampal GRs and thus is secondarily leading to loss of negative feedback of CORT secretion [69, 70]. In this context, it is interesting that hippocampus and amygdala exhibit differential expression of GR and MR [52, 71].

Cognitive decline associated with hippocampus has been extensively studied in respect to effects of stress (reviewed in [60, 72, 73]). However BLA, which is critical for generation and maintenance of fear and anxiety [74], has been relatively understudied in this regard [75, 76]. Future studies to bridge this gap will hopefully bring more clarity to biological mechanisms of stress inoculation.
